# Virome analysis and detection of ticks and tick-borne viruses in Shanghai, China

**DOI:** 10.3389/fmicb.2025.1699705

**Published:** 2025-10-22

**Authors:** Wenbo Zeng, Limin Yang, Lei Cui, Chuhan Liang, Dan Zhu, Yuan Fang, Yi Zhang, Hongxia Liu

**Affiliations:** 1 Ministry of Education Key Laboratory of Contemporary Anthropology, School of Life Sciences, Fudan University, Shanghai, China; 2 National Institute of Parasitic Diseases at Chinese Center for Disease Control and Prevention (Chinese Center for Tropical Diseases Research), Shanghai, China; 3 National Key Laboratory of Intelligent Tracking and Forecasting for Infectious Diseases, Shanghai, China; 4 Key Laboratory on Parasite and Vector Biology, National Health Commission, Shanghai, China; 5 WHO Collaborating Centre for Tropical Diseases, Shanghai, China; 6 National Center for International Research on Tropical Diseases, Ministry of Science and Technology, Shanghai, China; 7 Department of Vector Control and Prevention, Division of Infectious Diseases Control and Prevention, Shanghai Municipal Center for Disease Control and Prevention, Shanghai, China; 8 School of Global Health, National Center for Tropical Disease Research, Shanghai Jiao Tong University, Shanghai, China

**Keywords:** virome, tick-borne viruses, next-generation sequencing, tick surveillance, Shanghai, emerging infectious diseases

## Abstract

**Introduction:**

Ticks are well-known ectoparasites and vectors responsible for transmitting a diverse range of pathogens, including viruses, bacteria, and protozoa, many of which pose substantial risks to public health and livestock. In recent decades, the incidence and diversity of tick-borne diseases have increased globally, with several novel tick-borne viruses (TBVs) being discovered.

**Methods:**

This study aimed to characterize the virome of ticks collected from various locations in Shanghai, China, using next-generation sequencing (NGS). A total of 2,568 ticks belonging to three dominant species—*Haemaphysalis flava*, *Haemaphysalis longicornis*, and *Rhipicephalus sanguineus sensu lato*—were collected and analyzed through metagenomic sequencing.

**Results:**

The sequencing analysis identified 214 viral contigs classified into 32 viral families, including Chrysoviridae, Phenuiviridae, Partitiviridae, Nairoviridae, Dicistroviridae, Reoviridae, Botourmiaviridae, and Flaviviridae. Several TBVs with potential relevance to human and animal health, such as Cheeloo Jingmen-like virus (CJLV), Songling virus (SGLV), brown dog tick phlebovirus 1 (BDTPV1), brown dog tick phlebovirus 2 (BDTPV2), and Wuhan mosquito virus 1 (WMV1), were detected. Significant differences in virome composition among tick species based on geographical locations were also observed.

**Discussion:**

These findings highlight the influence of environmental factors on viral diversity in ticks and underscore the need for ongoing surveillance of TBVs. Implementation of longitudinal virome monitoring across tick developmental stages in Shanghai will provide critical insights for early warning systems, disease prevention strategies, and public health interventions.

## 1 Introduction

Ticks are ectoparasites that feed on the blood of vertebrates (Moran et al., 2008) and serve as vectors for a wide variety of pathogens, including viruses, bacteria, Rickettsia species, spirochetes, protozoa, mycoplasma, chlamydia, and Bartonella species (Dunaj et al., 2021; Luo et al., 2021; Pollet et al., 2020). Globally, ticks are second only to mosquitoes as vectors of infectious diseases that significantly impact public health and agriculture (Dantas-Torres et al., 2012). Tick-borne pathogens (TBPs) and associated diseases are prevalent worldwide, causing considerable harm to human health and economic productivity. For instance, babesiosis and theileriosis are among the most common parasitic blood diseases in tropical and subtropical regions, primarily transmitted between humans and animals through tick bites (Yu Zhijun, 2015). Ticks are temporary parasites, spending most of their life cycle detached from their hosts (Davari et al., 2017). After feeding, they drop off to molt or reproduce, with fertilized females laying eggs in their natural habitats (Haut et al., 2020). Approximately 94% of a tick’s lifespan is spent off-host, often within vegetation, where a substantial number of ticks exist freely (Davari et al., 2017).

A sharp rise in the number of tick-borne diseases has been observed in China over the past four decades, with the identification of numerous novel pathogens (Fang et al., 2015; Wang et al., 2019a). For example, the discovery of the severe fever with thrombocytopenia syndrome virus (SFTSV) in 2009, which caused multiple fatalities (Kodama et al., 2021; Liu S. et al., 2014; Xu et al., 2011), and the Alongshan virus in 2017, which exhibited clinical symptoms similar to the tick-borne encephalitis virus (TBEV) and was formally described in 2019 (Wang et al., 2019a). More recently, the Yezo virus, a new species of orthonairovirus, which causes multiple infections, was identified in Hokkaido, Japan (Kodama et al., 2021). As of 2018, at least 160 TBVs from 12 virus genera were identified, many of which pose a significant threat to human health. Some recent discoveries include novel TBVs such as Alongshan virus (ALSV), SGLV, Beiji nairovirus (BJNV), Heartland virus (HRTV), Tamdy virus, Tacheng tick virus-1 (TcTV-1), and Guertu virus (GTV). These findings highlight the presence of previously unknown TBVs associated with human diseases (Cai et al., 2023; Ma et al., 2021; McMullan et al., 2012; Meng et al., 2019; Shen et al., 2018; Wang et al., 2021; Wang et al., 2019a; Zhang et al., 2021; Zhou et al., 2019). However, effective vaccines for most tick-borne diseases remain unavailable, emphasizing the critical need for enhanced surveillance and control measures to enable early detection, prediction, and prevention.

Next-generation sequencing (NGS) technology offers a transformative approach to pathogen detection by enabling the direct sequencing of all genetic material in environmental samples without the need for metagenomic libraries (Suenaga, 2012). NGS, or high-throughput sequencing, features unparalleled speed, high accuracy, and cost-effectiveness. This technology has been extensively utilized in genome sequencing and related fields, creating new possibilities for pathogen detection. Virome analysis, a specific application of NGS (Goodwin et al., 2016), enables the sequencing of all viral nucleic acids present in a sample (Forterre, 2017). Despite its advantages, the process presents challenges such as complex sample preparation processes, high cost of data analysis, and difficulty in identifying viruses (Forterre, 2017; Gomard et al., 2021; Zhao et al., 2020). Tick virome studies have revealed an abundance of diverse viruses, including novel and highly divergent species that infect vertebrates, invertebrates, and plants (Xia et al., 2015; Zhao et al., 2020). Comparative studies indicate a significant difference in viral diversity among ticks, with evidence suggesting that geographic location, rather than natural host factors, plays a major role in shaping viral families (Zhao et al., 2020). Tick microbiota varies considerably between sexes and across geographic regions.

While northern China has been extensively surveyed for TBVs (e.g., SFTSV), the virome composition of ticks in Shanghai, a megacity with a high human-animal-vector contact rate, remains uncharacterized. This represents a significant blind spot in regional emerging disease surveillance. Tick-borne diseases and tick bites are common in regions surrounding Shanghai, posing serious health risks to local populations. Between 2009 and 2022, an increasing number of severe fever with thrombocytopenia syndrome (SFTS) cases were reported in 23 provinces (Chen et al., 2022; Liu Q. et al., 2014; Zhan et al., 2017; Zhang and Xu, 2016). Shanghai, located in the alluvial plain of the Yangtze River Delta, features soft soil, a low and flat topography, and a warm, humid subtropical monsoon climate with distinct seasons. These natural conditions create a highly favorable environment for tick proliferation (Zeng et al., 2022). Shanghai, as one of the largest international metropolitan areas in the world, is characterized by a dense human population, extensive urban–rural interfaces, and a high level of human-animal contact through pet ownership, livestock trade, and urban wildlife. These conditions create a favorable ecological niche for ticks and increase the opportunities for cross-species transmission of pathogens. Given its role as a global transportation hub, the emergence or introduction of novel TBVs in Shanghai would not only pose a direct threat to local public health but also carry the risk of wider regional or even international dissemination. Therefore, characterizing the virome of ticks in Shanghai provides essential baseline data to understand potential zoonotic risks and strengthen early warning and prevention systems in this highly interconnected urban environment. In this study, we collected ticks from Shanghai and utilized NGS to obtain RNA sequencing data, offering valuable insights into the diversity of TBVs and their associated public health risks.

## 2 Materials and methods

### 2.1 Sample collection and identification

**Figure 1:**
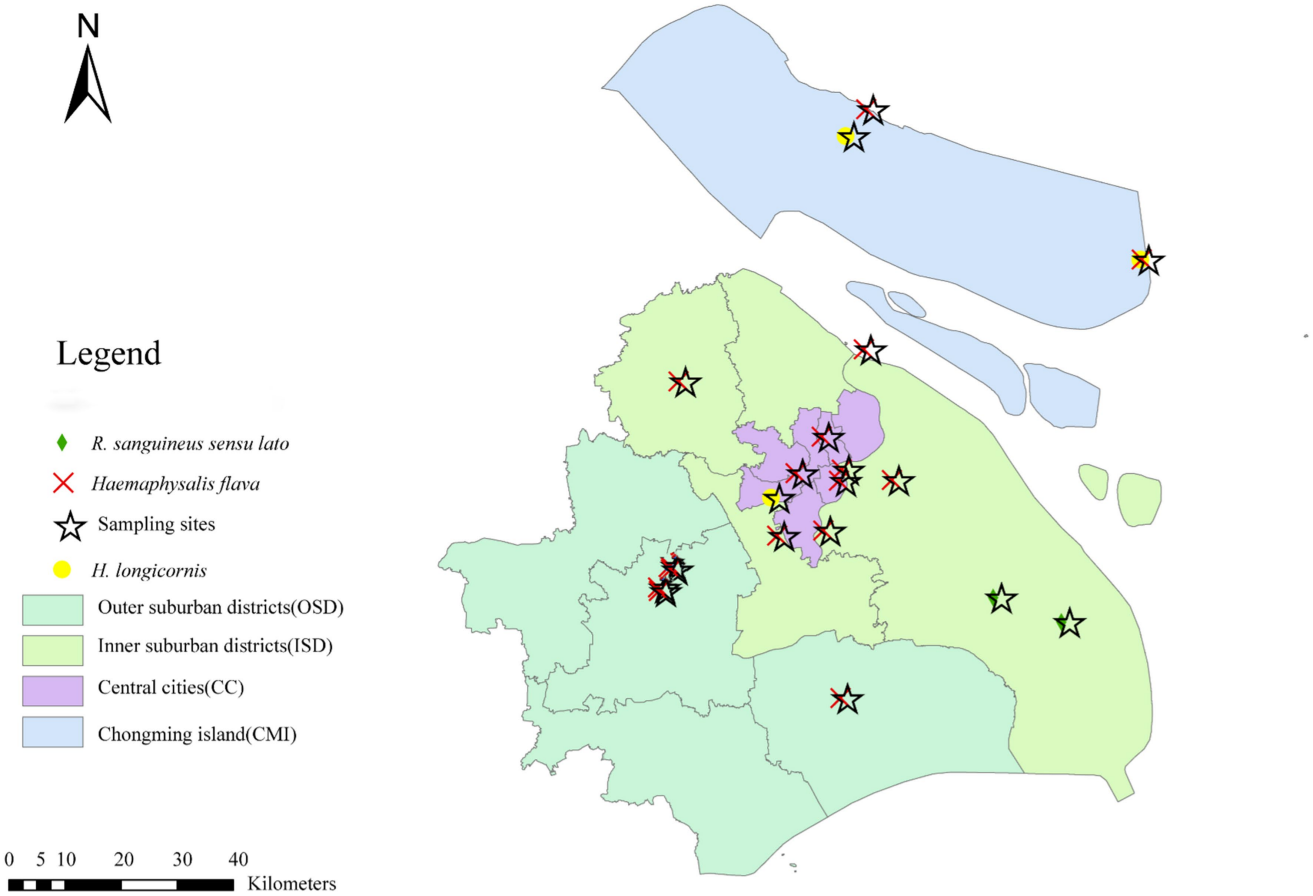
Spatial distribution of tick surveillance sites and tick species in Shanghai, China.

These ticks were collected from 19 sampling sites in Shanghai (Figure 1), and the sample points were selected from random sites in each functional area of the city. Questing ticks were collected by dragging flags on the vegetation layer during the day. In addition, parasitic ticks were collected from animals (dogs, goats, sheep, cows, etc.). First, different morphological characteristics were observed by an entomologist, Zhu Dan, to identify the species and development stages of the collected ticks (Yamaguti et al., 1971), followed by identification of cytochrome C oxidase subunit I (COI) (Abdullah et al., 2016) and 16S rDNA (Black and Piesman, 1994) genes to further determine the species of ticks (Zeng et al., 2022). All ticks from each site were included in the study. Second, in the lab stage, the ticks were rinsed with 75% ethanol for 1 min to remove any environmental contaminants, followed by rinsing with deionized water for 5 min to remove 75% ethanol, and finally stored in a refrigerator (−80 °C).

### 2.2 Preparation of ticks and extraction of viral RNA

Tick tissue samples were homogenized in TRIzol with steel beads using an automatic grinder. Homogenates were centrifuged at 15,000 g for 30 min at 4 °C, and the supernatant was transferred to a 96-well plate for RNA extraction using the MagNA Pure 96 System (Roche). The total extracted RNA was eluted in RNase-free water and stored at −80 °C. Aliquots were used for RNA quantification and reverse transcription, and agarose gel electrophoresis was used to assess the quality of RNA. For virome sequencing, 30 μL of RNA per sample was submitted to Meg Biotechnology Co., Ltd. (Guangzhou, China).

### 2.3 cDNA library preparation for NGS

The whole transcriptome was amplified using the QIAGEN kit (150,054 REPLI-g cell WGA & WTA kit), and the amplification products were tested using Thermo NanoDrop One, Life Technologies Qubit 4.0, and 1% agarose electrophoresis. Sequencing libraries were generated using ALFA-SEQ DNA library prep kit for Illumina (FINDROP, Guangzhou) following the manufacturer’s recommendations, and index codes were added. The library quality was assessed on the Qubit dsDNA HS Assay Kit (Life Technologies, Grand Island, NY) and Bioptic Qsep400 system. Finally, the library was sequenced on Illumina NovaSeq 6,000, and 150 bp paired-end reads were generated.

### 2.4 Next-generation sequencing

The raw data was processed using SOAPnuke (v1.5.6) to acquire the clean data for subsequent analysis. Using BWA software (v0.7.17, parameter: mem –k 30) to compare clean reads with ribosome database (Silva.132) and host database, respectively, the result that the comparison length was less than 80% of the total length of reads was filtered, and the host sequence was removed. The BWA software (v0.7.17, parameter: mem-k 30) was also used to compare clean reads to the virus reference database (Virus-NT), and results with comparison length less than 80% of the total length of reads for preliminary virus classification were filtered. Megahit software (v1.1.2, parameter: --presets meta-large, −-minimum-contig-length 300) was used to assemble clean data, and BWA software (v 0.7.17) was used to compare clean reads with assembly results to calculate the utilization ratio of reads. The sequences that were compared to the host sequence database using Basic Local Alignment Search Tool (BLAST) software (v2.9.0 +) were removed. If there was more than one sample, CDHIT software (v4.7, parameter: -c 0.95 -aS 0.8) was used to cluster the contigs of all samples to obtain a unique contig. Using BLAST software (v2.9.0 or later), the unique contigs were compared against a virus-specific database (separated from the NT database). Contigs with an alignment similarity of ≥80% and an alignment length of ≥500 bp were considered significant. Contigs were compared with virus databases from nucleotide (NT), non-redundant (NR), and Hidden Markov Model (HMM) profiles (vpfs and vfam), and the combined results were taken as candidate virus sequences. These candidate virus sequences were compared to National Center for Biotechnology Information (NCBI) taxonomy database, and if more than 20% of the first 50 alignment results that supported the sequences were non-viral sequences, the candidate virus sequences were excluded and the rest were considered as new virus sequences.

### 2.5 Virus screening

The RNA was reverse transcribed using a reverse transcription kit (TaKaRa, China) in accordance with the manufacturer’s instructions. Nested reverse transcription-polymerase chain reaction (RT–PCR) was used to confirm and amplify the viral genomes of the original samples. PCR primer pairs were designed from contigs using Primer Premier 5.0 (Premier Biosoft International, United States). The primers used to amplify viral sequences have been listed in Supplementary Table S1. The 25-μL reaction volume included 2 × Master Mix (Tiangen, China) 12.5 μL; ddH2O 9.5 μL; forward primer (10 pmol/μL) 1 μL; reverse primer (10 pmol/μL) 1 μL; and DNA or cDNA template 1 μL. The cycling conditions were as follows: 35 cycles (outer PCR) or 40 cycles (inner PCR) of denaturation at 94 °C for 30s; annealing at 56 °C (or adjusted in accordance with primer pairs) for 30 s; and extension at 72 °C for 1 min. Double-distilled water replaced cDNA in the negative control. All PCR products were analyzed by 1% agarose gel electrophoresis, and positive products were sequenced using Sanger sequencing.

### 2.6 Phylogenetic analysis

The obtained NT sequences and viral contigs were compared with those available in GenBank using the NCBI (Bethesda, MD, United States) BLAST search engine^1^, and multiple sequence alignments were performed using the MEGA X (version 10.0) multiple alignment tool with the default parameters in MEGA X. The phylogenetic maximum likelihood (ML) estimation was performed using MEGA X, and the ML tree was constructed. The LG model, a substitution model, was used to describe amino acid replacement patterns in protein sequences, while the gamma distribution was applied to account for among-site rate heterogeneity, reflecting the variation in evolutionary rates across different positions within the protein. Bootstrap values were estimated for 1,000 replicates.

### 2.7 Analysis and visualization

According to the comparison results between the virus contigs and the virus NT database by BLAST (v2.9.0+), the best hit with e < 1e-5 was selected for species annotation, and the results without comparison were expressed using NA. The BWA software (v0.7.17, parameter: mem –k 30) compared clean reads after removing the host with virus contigs, filtered the comparison results that measured less than 80% of the total length of reads, counted the proportion of virus reads and the distribution of virus reads according to the annotation results of virus contigs, and, in the end, calculated the reads per kilobase million (RPKM) of each virus contig.

Metagenemark (v3.38) was used to predict the gene sequence of the virus contigs and evaluate the predicted gene number, length, etc.

The protein sequence of the gene was compared to the virus sequence in UniProtKB/Swiss-Prot database^2^ by using BLASTp software (v2.9.0+), and the best hit comparison result of e < 1e-3 was screened to obtain the virus function information.

The abundance and diversity of environmental viruses were reflected in the analysis of single sample diversity (alpha diversity). The results were presented using Shannon index, considering the species richness and evenness. The difference in species composition of various samples was compared through beta diversity analysis, and Bray–Curtis distance matrix was used to obtain the sample clustering heat map and principal coordinate analysis (PCoA) or principal component analysis (PCA) plot.

LDA effect size (LEfSe) tool was used to identify the biomarker of each group based on RPKM of virus sequence. First, non-parametric factorial Kruskal–Wallis (kw) sum rank test was used to detect virus contigs with significant difference in abundance among different groups. Second, Wilcoxon rank sum test was used to judge the difference between the two groups. Finally, linear discriminant analysis (LDA) was used to evaluate the impact of significant virus (LDA score) and the biomarkers obtained in different groups. If there were only two groups, Student’s *t*-test, Wilcox rank sum test, and Wilcoxon signed rank test or Welch’s *t*-test were used in R software to evaluate and correct through false discovery rate (FDR). However, in case of more than two groups, Kruskal–Wallis rank sum test or one-way ANOVA was used to evaluate through R software and correct through FDR.

CRISPR-CAS spacer database was constructed from bacterial genome sequences in RefSeq database using CRISPR Recognition Tool^3^. Using blastn-short (v2.9.0+) to compare the identified viruses with CRISPR CAS spacer database, the best hit was selected as the possible host information of phage when the e-value < 1e-10, the comparison similarity was more than 95%, and the coverage of spacer was more than 80%.

### 2.8 Specific PCR for detection of some pathogens in ticks

Based on the results of 16S rDNA gene amplicon sequencing, genus-/group-specific PCR was performed to confirm the presence of TBPs in individual ticks. PCR was performed using the PCR System 9,700 (Applied Biosystems, GeneAmp®, United States). For PCR, 2 μL of each DNA sample (150–330 ng) was used as the template for the first round, and 1 μL of the primary PCR production was used as the template for the second round. For the first round, a negative control (ddwater) and an extraction control mentioned above were included in each PCR experiment. Tube strips with individual caps were used in amplification steps to prevent cross-contamination, and all PCR amplifications were carried out using PrimeSTAR® HS (Premix) (TaKaRa, Beijing, China). All operations were carried out in a biological safety cabinet. Amplified products were then electrophoresed in 1.5% agarose gel, and the positive amplicons were sent to TSINGKE Biological Technology (Beijing, China) for sequencing. The PCR primers for Crimean–Congo hemorrhagic fever virus (CCHFV), Dabie bandavirus (DBV), TBEV, South bay virus (SBV), Nairobi sheep disease virus (NSDV), Hantaan virus (HTNV), West Nile virus (WNV), Japanese encephalitis virus (JEV), Dabieshan virus (DBSV), Alongshan virus (ALSV), and Jingmen virus (JMV) are presented in Supplementary Table S1.

## 3 Results

### 3.1 Tick collection, classification, distribution, and phylogenetic analysis

A total of 2,568 hard ticks were morphologically identified as *H. flava* (*n* = 2,102; 80 adults, 295 nymphs, and 1,727 larvae), *H. longicornis* (*n* = 151; 65 adults, 8 nymphs, and 78 larvae), and *R. sanguineus sensu lato* (*n* = 315; 231 nymphs and 84 adults). These identifications were subsequently confirmed by species-specific PCR and sequencing.

BLAST comparison of partial 16S rDNA and COI gene sequences from *H. longicornis*, *H. flava*, and *R. sanguineus sensu lato* depicted the highest similarity (98–100%), with the corresponding sequences of each species available in GenBank.

Results of the phylogenetic analysis of 16S rDNA sequences for these three tick species (i.e., *H. flava*, *H. longicornis*, and *R. sanguineus sensu lato*) were consistent with their morphological identifications. Specifically, *H. flava*, *H. longicornis*, and *R. sanguineus sensu lato* clustered with reference sequences of *H. flava* (KP324926, MW064043, and MH520707; Figure 1); *H. longicornis* (MW773214; Supplementary Figure S1); and *R. sanguineus sensu lato* (MT322611, MH481870, OL757514, MW800155, KC170744, and MG651947; Supplementary Figure S2), respectively. Further phylogenetic analysis based on COI gene sequences exhibited that *H. flava* and *H. longicornis* grouped within the same clades as reference *H. flava* (MW066331, JQ737097, MN784164, and MN650208) and *H. longicornis* (JQ737092, MT465132, KM821501, KF284075/KF284074, and KF284073; Supplementary Figure S3). Likewise, *R. sanguineus sensu lato* formed a clade with other *R. sanguineus sensu lato* reference sequences (HM193874, MG969507, MZ401443, HM193b73, and JQ737084) in the COI tree (Supplementary Figure S4).

### 3.2 Tick virome profiling

A total of 388,779,089 reads were obtained from the sequencing. On average, each pool yielded 48,597,386 reads (SD = 4,723,352), and after quality trimming, the average read count was 18,961,484 (SD = 7,141,198). Following comparison to tick genomes in the NCBI databases and removal of tick-derived sequences, as well as comparison of the remaining clean reads to the virus reference database (Virus-NT), each pool retained an average of 38,729 (SD = 67,133) reads of viral origin. Reads from each pool were independently assembled into contigs, producing on average 32,232 (SD = 5,618) contigs. Contigs matching host sequences were then removed, leaving an average of 29,527 (SD = 7,297) virus-specific contigs (Table 1).

**Table 1:** Tick pools, reads, and contigs and virus contigs information recovered through the bioinformatic analysis.

Pool	Sample code	Sampling site and species	Number of ticks	Raw reads	Cleaned reads	Virus reads	Contigs	Virus contigs
1	CCF	*H. flava*	56	51,451,235	20,198,830	11,560	26,556	26,421
2	CCL	*H. longicornis*	22	44,447,140	18,348,437	8,895	28,230	28,135
3	CMIF	*H. flava*	1,184	55,002,181	35,340,834	1,242	31,056	31,039
4	CMIL	*H. longicornis*	129	51,182,099	20,156,123	16,038	32,360	32,246
5	ISDF	*H. flava*	38	52,790,028	14,845,622	203,192	41,776	41,772
6	OSDF1	*H. flava*	223	46,522,821	15,339,477	17,809	34,358	34,295
7	OSDF2	*H. flava*	601	41,133,722	14,274,581	34,667	25,626	25,547
8	OSDS	*R. sanguineus sensu lato*	315	46,249,863	13,187,968	16,429	37,892	16,761

In the subsequent analysis, any contigs identified as DNA viruses were excluded, while contigs identified as RNA viruses or those of uncertain viral type were retained. This process revealed 37 contigs classified as confirmed RNA virus contigs and 488 contigs classified as suspected virus contigs (Supplementary Figure S5). Among these, sequences in both the novel and confirmed categories were retained, resulting in a final set of 214 RNA virus contigs. A length distribution plot (Supplementary Figure S6) shows that 61.21% of these viruses are under 1,000 bp long, with some exceeding 5,000 bp. The 214 RNA virus contigs were classified into 32 virus families (Supplementary Figure S7), which include Chrysoviridae, Phenuiviridae, Partitiviridae, Nairoviridae, Dicistroviridae, Reoviridae, Botourmiaviridae, and Flaviviridae.

### 3.3 Analysis of tick virus community structure in different geographical populations

At the family level, the abundance of uncharacterized viruses is relatively high in most groups, and Flaviviridae, Nairoviridae, and Coronaviridae are present in every group. However, the dominant virus family varies across groups (Supplementary Figure S8).

At the genus level, unknown and unclassified viruses also show high abundance in all groups, and both Pestivirus and Orthonairovirus are present in each group. However, the dominant genera differ among these groups (Supplementary Figure S9).

### 3.4 Diversity analysis of tick virus community in different geographical populations

The difference in the Shannon index is relatively small, indicating that species richness and evenness are essentially similar (Supplementary Table S2). Meanwhile, the PCoA plot shows that the viral composition in ticks from Shanghai primarily varies according to both tick species and geographical location (Supplementary Figure S10).

### 3.5 Jingmen tick virus group

**Figure 2:**
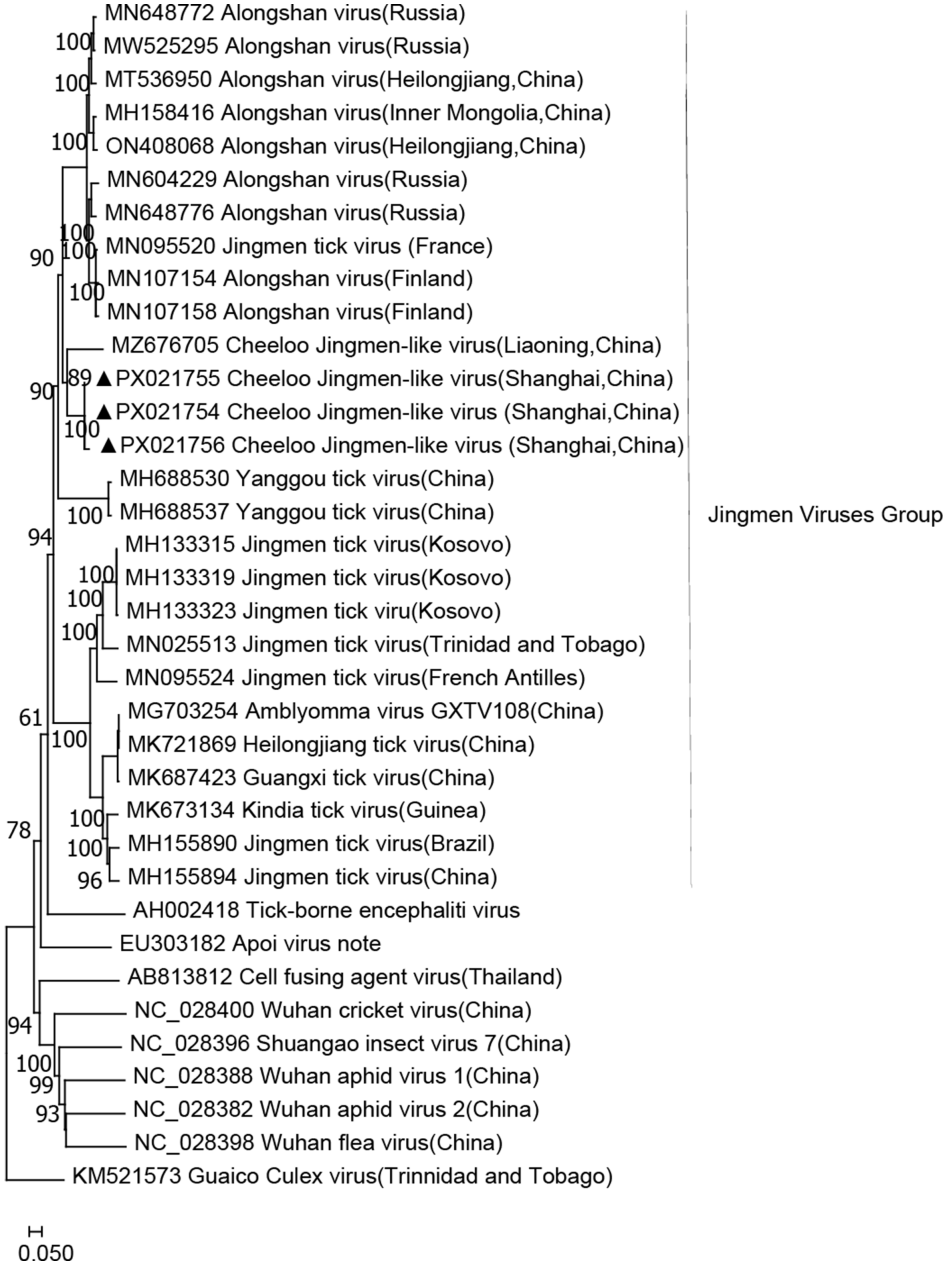
Phylogenetic tree of CJLV based on the nucleotide sequences of segment two. The sequence obtained in this study is indicated with a black triangle.

Jingmen tick virus (JMTV) group has a segmented RNA genome, believed to be derived from a non-segmented flavivirus, and is widely distributed in the tick population in China. The PCR screening of all samples using specific primers targeting segment two revealed that the infection rate of CJLV in *H. longicornis* samples was 33.33% (5/15). As a result, only CJLV (33.33%, 5/15) was detected in *H. longicornis* from Shanghai. CJLVs identified in *H. longicornis* were shown to be clustered with CJLV (MZ676705; Figure 2). However, CJLV was not detected in *H. flava* or *R. sanguineus sensu lato*. The gene fragments identified by metagenomic analysis ranged from 300 to 1,068 bp in size. These JMTV fragments exhibited the highest similarity to the corresponding CJLV sequences in GenBank, sharing 68.31–75.39% identity with the closest matches (Figure 2).

### 3.6 Orthonairovirus

**Figure 3:**
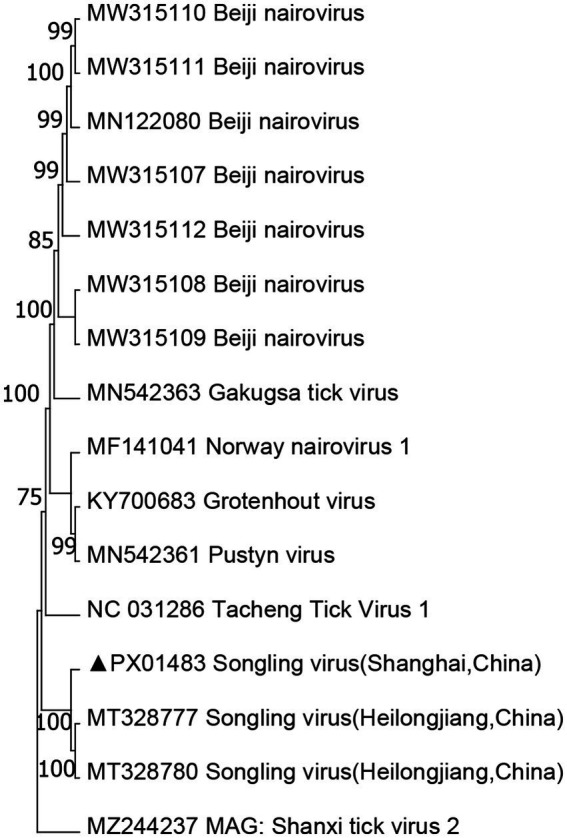
Phylogenetic tree of SLV based on the nucleotide sequences of segment S. The sequence obtained in this study is indicated with a black triangle.

Nairoviridae belongs to the Bunyaviridae family and comprises 12 species, many of which are TBVs. Orthonairovirus is a genus within Neroviridae that includes important TBPs such as CCHFV and NSDV, along with several other viruses of uncertain characteristics found in ticks, birds, and mammals. The SGLV has a genomic structure and morphological features similar to those of the putative Nello virus. It forms a unique evolutionary branch within the Tamdy orthonairovirus group of the Nello virus family. In this study, the Songling virus fragments showed the highest similarity to SGLV sequences in GenBank, sharing 70.40–72.95% of identity with the closest matches. Phylogenetic analyses (Figure 3) revealed that all three identified viruses clustered with the other SGLV. Moreover, SGLV was detected in *H. flava* and *H. longicornis* samples.

### 3.7 Prevalence of tick-borne pathogens in individual pools

Around 2,102 *H. flava* were divided into 211 pools, 151 *H. longicornis* were divided into 15 pools, and 315 *R. sanguineus sensu lato* were divided into 32 pools. Notably, ALSV exhibited strict host tropism for *H. longicornis*, mirroring patterns observed in Chongming Island(CMI) regions. In contrast, the novel JMV-like viruses expressed broader host range across Rhipicephalus and Ixodes species, implying differential transmission dynamics that warrant further vector competence studies.

### 3.8 Uukuvirus

**Figure 4:**
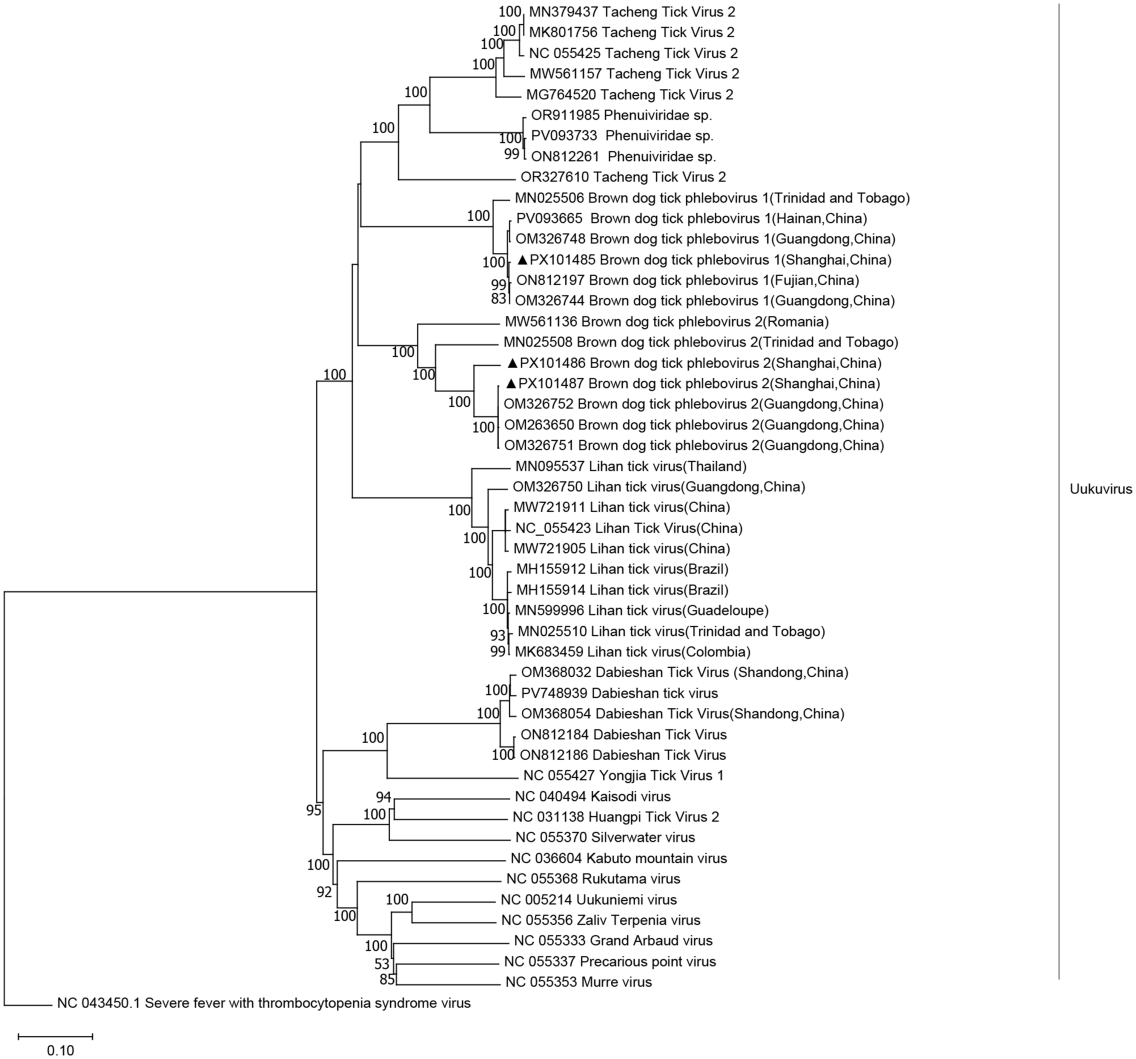
Phylogenetic trees were constructed based on the L segment nucleotide sequences of representative viruses in the genus *Uukuvirus*. The sequence obtained in this study is indicated with a black triangle.

We obtained three complete viral genomes, which were designated as BDTPV1 and BDTPV2. BDTPV1 comprised two complete genomes, each containing two segments, large (L) and small (S), encoding the putative RNA-dependent RNA polymerase (RdRp) and nucleoprotein, respectively. BDTPV2 comprised a single complete genome, also consisting of L and S segments encoding RdRp and nucleoprotein. The length of the L and S segments for BDTPV1 were 6,530 bp and 1,343 bp, sharing 96.5% nucleotide identity in the RdRp with BDTPV1, which has been reported in Trinidad and Tobago. The length of L and S segments for BDTPV2 was 6,491 bp and 1,960 bp. BDTPV2 GD shared 96.7% nucleotide identity in the RdRp with BDTPV2, which has been reported in Guangdong, China. Interestingly, both BDTPV1 and BDTPV2 were found to co-exist in *H. flava*. Phylogenetic analyses (Figure 4) revealed that all three identified viruses clustered with the other bunyaviruses lacking the M segment and shared a common ancestor with the Uukuvirus group. They were genetically related to those identified in Guangdong (China), Hainan (China), and Trinidad and Tobago.

### 3.9 Orthophasmavirus

**Figure 5:**
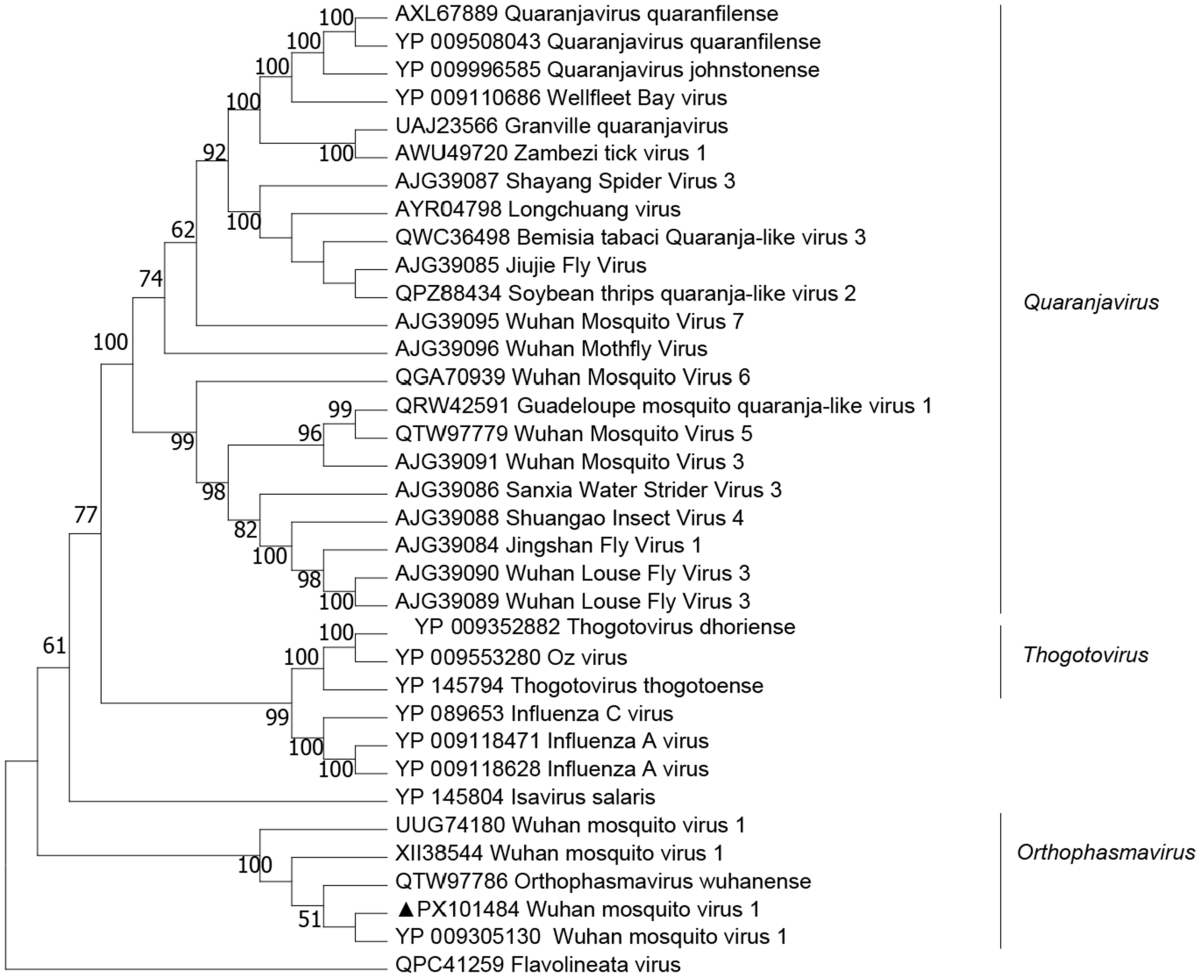
Phylogenetic trees of Wuhan mosquito virus 1 (WMV1) were constructed based on the RNA-dependent RNA polymerase (RdRp) protein sequences of representative viruses.

We obtained two viral genomes designated WMV1. The genomes of these viruses contained bi-segments, large (L) and small (S) segments, encoding the putative RdRp and nucleoprotein, respectively. The lengths of L and S segments for WMV1 were 4,786 bp and 1,430 bp, respectively, and they shared 98.1–98.3% amino acid identity in the RdRp with WMV1, which has been reported in China (YP_009305130) (Figure 5).

## 4 Discussion

The morphological identification of all the samples was confirmed by both COI and 16S rDNA (Lv et al., 2014). A 650 bp segment versus a 410 bp region may include different informative sites and result in changes in tree topology. The inclusion or exclusion of certain GenBank entries changes the comparative framework, and the addition of divergent outgroups or close relatives can reorder internal branches (Aly, 2014; Hornok et al., 2017). Gap penalties and trimming of poorly aligned ends alter the homology assessment (Aly, 2014; Ashkenazy et al., 2019; Steenwyk et al., 2020).

Despite the low detection rate, multiple viruses were identified using NGS, with confirmation possible via PCR and sequencing. When viral load is low (<10^3^ copies/μL), host rRNA and mRNA can dominate the library. Hence, even after rRNA depletion, only a few dozen to a few hundred viral reads are recovered per sample (Li et al., 2025). Among these, only one virus, the CJLV, was successfully verified through segment-specific RT-PCR in this study. Several factors explain this outcome: the primer sequences used lacked sufficient specificity for the targeted viruses or the virome analysis required further optimization. Furthermore, variations in sample size and collection timing across regions may influence the observed viral prevalence (Cai et al., 2023). The study aimed to obtain baseline information on TBVs circulating in Shanghai, which provides useful data for future diagnosis, prevention, and control of tick-borne diseases in this region. For this reason, the sequencing pools contained a mixture of larvae, nymphs, and adults, not just adult ticks. It is recognized that larval ticks may carry different viral profiles compared to nymphs and adults because of their limited host-feeding history. Nevertheless, including larvae ensured the dataset covered all developmental stages in the local environment, thereby capturing viruses that may be vertically transmitted or persist from the earliest stages.

Provisional presence of JMV was identified in *H. longicornis* ticks in China. In a 2024 virome survey, CJLV was among the most prevalent viruses across both genetic clades of *H. longicornis* (Ye et al., 2024). Its glycoproteins VP1a/VP1b share ~91–100% and ~92–100% amino acid identity, respectively, with ALSV from *H. longicornis*, and it clusters within the JMV group (Ye et al., 2024). ALSV, a newly discovered segmented TBV within the *Flaviviridae* family, was linked to 86 cases of febrile illnesses in Heilongjiang Province and Inner Mongolia Autonomous Region in 2019 (Wang et al., 2019b). Some JMTV and ALSV have been found in febrile patients; however, to date, there is no specific evidence that CJLV causes disease in humans (Liu et al., 2024). Although genetically distinct from JMTV, CJLV is more closely related to JMTV than to other related jingmenviruses. JMTV, initially isolated from *H. longicornis* ticks in China, has a broad host range, including cattle, dogs, and goats. Variants of JMTV have also been identified in Brazil, Uganda, and the United States, suggesting a more widespread distribution than previously estimated (Liu et al., 2022). In our tree based on segment two (NS2) sequences, the Shanghai-derived CJLV sequences fall into the same clade as the Liaoning Province isolates (e.g., GenBank MZ676705), which are from northeastern China, with high bootstrap support (>90%). Interestingly, the collection environment for *H. longicornis* ticks overlaps with the habitat of *Cervus nippon* in northeastern China. As *C. nippon* has been introduced as an ornamental species and allowed to grow freely in these habitats, it is plausible that the movement of the Cervus population contributed to the presence of the virus in Shanghai, where a tick-borne transmission cycle may have been established within the park ecosystem. We recommend enhanced national surveillance of TBVs during animal translocation and a comprehensive investigation of domestic animals, wildlife, and associated tick vectors in Shanghai, China.

The SGLV has been detected in patients hospitalized after tick bites in Heilongjiang Province and Inner Mongolia (Ji et al., 2023). However, there is limited information on the *Nerovirus* genus, including SGLV, carried by wild animals and livestock in East China. In particular, studies on the isolation and culture of these viruses in Shanghai are lacking. Future efforts should focus on monitoring and researching TBPs in wild and domestic animals and their associated tick vectors within Shanghai.

BDTPV1 had a prevalence rate of 78% of *R. sanguineus* ticks and was also found in two R. microplus ticks (<1%) (Sameroff et al., 2019). BDTPV2 had the highest similarity (93% amino acid in the polymerase) to tick phlebovirus identified in R. bursa ticks in Turkey (Dinçer et al., 2017). In Guangdong, China, both BDTPV1 and BDTPV2 were found to co-exist in *R. sanguineus* (Guo et al., 2022). However, in our study, BDTPV1 and BDTPV2 were found in *H. flava*. These findings broaden the known host range of BDTPVs and highlight ecological plasticity across tick genera. Monitoring co-circulation and potential reassortment is warranted. Vector competence experiments, serosurveys in domestic animals, and metatranscriptomics of additional tick species will clarify transmission dynamics, pathogenic potential, and regional risk for humans and the livestock population.

RNA sequencing of 70 arthropod species collected from across China led to the first identification of WMV1. Researchers characterized its three-segmented, negative-sense RNA genome and proposed it as a novel mosquito-associated bunyavirus lineage (Li et al., 2015; Shi et al., 2016). Monthly meta-transcriptomic monitoring of urban and suburban mosquitoes highlighted that WMV1 and Xincheng anphevirus dominated *Anopheles* and *Culex* populations, displaying marked seasonal swing and hinting at a “core mosquito virome.” Subsequent phylogenetic analyses grouped WMV1 with related viruses in the newly created family Phasmaviridae, genus *Orthophasmavirus*, confirming that it is generally restricted to mosquitoes. Virome analysis of *Anopheles sinensis* from Zhoushan Island (Zhejiang, China) recovered a near-complete WMV1 genome, indicating that the virus circulates in both coastal islands and inland regions of China (Yang et al., 2023). In the present study, WMV1 was detected in *H. flava* ticks. The sequences cluster closely with previous WMV1 isolates, suggesting that *H. flava* can carry the virus. WMV1 is typically recognized as a mosquito-borne virus. Ticks were washed with 75% ethanol and sterile water prior to RNA extraction, reducing surface contamination. We obtained complete L and S genomic segments of WMV1 from *H. flava*, which strongly supported that the detection was genuine rather than contamination, and this was a significant finding. This underscores the need for cross-host studies to unravel the transmission dynamics of WMV1. TBV transmission typically occurs during feeding and is activated by tick saliva molecules, as seen with TBEV and Powassan virus (POWV). Generalist tick species (e.g., *H. longicornis*) pose elevated zoonotic risk due to their catholic host preferences and demonstrated capacity for bridging sylvatic/domestic transmission cycles. However, several challenges remain in understanding virus host ecology (Xu et al., 2021), including. Viruses are heterogeneously distributed within their hosts, necessitating large sample sizes to accurately characterize the structure and composition of tick-borne virus (TBV) populations. Many unknown viruses exist in nature, and the classification of highly divergent viruses poses substantial challenges. Environmental variables significantly impact virus–host models, complicating comparisons of parasitic species across different geographic regions.

The observed diversity of tick species may result from variations in sampling times, climates, ecological environments, or human activities (Esteve-Gassent et al., 2016). The average residual viral read per NGS sample was 38,729 (SD = 67,133), and the average number of viral contigs was 29,527 (SD = 7,297), both of which were lower than comparable studies. Potential factors for these observations include higher initial sample storage temperatures (−20 °C), varying sample preparation protocols, and variations in sequencing platforms (Meng et al., 2019). Initial storage at −20 °C before transfer to −80 °C may lead to viral degradation. Moreover, the cDNA library preparation kits and sequencing platforms used may affect viral detection rates (Meng et al., 2019).

In addition, increased sequencing depth and sensitivity revealed microbial contamination in the RNA-seq library. Contaminants may have been introduced during any step from sample collection to sequencing. Despite washing ticks with PBS before RNA extraction, microbial species related to the ticks’ surfaces can remain due to environmental factors (Chandra et al., 2021). RNA-seq data also helps identify pathogenic fungi and eukaryotes. This study uncovered viral diversity in ticks similar to that found in Ixodes species and identified previously known viruses with pathogenic potential. However, no DNA viruses were detected, necessitating further investigation. A more systematic approach is recommended to comprehensively study the microbiota of Ixodes, identify potential pathogens, and refine methods for microbiota and virome research.

NGS technology has led to the discovery of numerous new viruses in *Ixodes* ticks, some of which have been linked to pathogenicity, such as the Arctic *Luonai* virus. A similar case involved ALSV, which belongs to the JMV group and included Mogiana tick virus (MGTV), Kinda tick virus (KITV), and Guangxi tick virus (GXTV). These findings underscore the importance of studying tick viromes for understanding their pathogenicity (Cai et al., 2023; Liu et al., 2022; Meng et al., 2019) and public health significance.

## 5 Conclusion

This study analyzed the viromes of three dominant tick species in Shanghai—*H. flava*, *H. longicornis*, and *R. sanguineus sensu lato*—using a metagenomic approach. Beyond investigating viral diversity, the research identified several TBVs with potential relevance to human and animal health, including CJLV, BDTPV1, BDTPV2, WMV1, and SGLV. While the genetic similarities between the newly discovered viruses and known human pathogens provide valuable initial insights, they are insufficient to conclusively establish the local transmission dynamics of these viruses. Nevertheless, the findings serve as a critical foundation for more detailed investigations into tick-borne virus ecology in Shanghai and support the development of evidence-based strategies for controlling the spread of viral diseases in urban environments.

## Data Availability

The data presented in this study are deposited in the China National Center for Bioinformation (CNCB) repository under the accession number PRJCA047529.

## Footnotes

^1^http://blast.ncbi.nlm.nih.gov/blast.cgi, accessed on 30 January 2025.

^2^ViralZone, https://viralzone.expasy.org/.

^3^CRT; http://www.room220.com/crt/.
