# Correction: Virome analysis and detection of ticks and tick-borne viruses in Shanghai, China

**DOI:** 10.3389/fmicb.2025.1731347

**Published:** 2025-11-10

**Authors:** Wenbo Zeng, Limin Yang, Lei Cui, Chuhan Liang, Dan Zhu, Yuan Fang, Yi Zhang, Hongxia Liu

**Affiliations:** 1 Ministry of Education Key Laboratory of Contemporary Anthropology, School of Life Sciences, Fudan University, Shanghai, China; 2 National Institute of Parasitic Diseases at Chinese Center for Disease Control and Prevention (Chinese Center for Tropical Diseases Research), Shanghai, China; 3 National Key Laboratory of Intelligent Tracking and Forecasting for Infectious Diseases, Shanghai, China; 4 Key Laboratory on Parasite and Vector Biology, National Health Commission, Shanghai, China; 5 WHO Collaborating Centre for Tropical Diseases, Shanghai, China; 6 National Center for International Research on Tropical Diseases, Ministry of Science and Technology, Shanghai, China; 7 Department of Vector Control and Prevention, Division of Infectious Diseases Control and Prevention, Shanghai Municipal Center for Disease Control and Prevention, Shanghai, China; 8 School of Global Health, National Center for Tropical Disease Research, Shanghai Jiao Tong University, Shanghai, China

**Keywords:** virome, tick-borne viruses, next-generation sequencing, tick surveillance, Shanghai, emerging infectious diseases

Affiliation information has been updated to ensure consistency with the following authors' institutional appointments at the time of the study: Yuan Fang^2,3,4,5,6,8^, Yi Zhang^2,3,4,5,6,8*^, Hongxia Liu^7*^

The original article has been updated.

