# Supplementary material for: Virome analysis and detection of ticks and tick-borne viruses in Shanghai, China
Source: Front Microbiol. 2025 Oct 22;16:1699705. doi: 10.3389/fmicb.2025.1699705 (PMC12586129; doi:10.3389/fmicb.2025.1699705)
Supplement: Supplementary file 1 [file Presentation_1.zip › Supplementary_Material.docx]

Supplementary Material

**Table S1.** Primers used in this study.

| **Target** |  | **Primer name** | **Sequence(5^’^-3^’^)** | |  |
| --- | --- | --- | --- | --- | --- |
| CCHFV |  | CCHFF2 | tggacaccttcacaaactc | | [1] |
|  |  | CCHFR3 | gacaaattccctgcacca | 530bp |  |
|  |  | CCHFF3 | gaatgtgcatgggttagctc | |  |
|  |  | CCHFR2 | gacatcacaatttcaccagg | 220bp |  |
| DBV |  | NP-2F | catcattgtctttgccctga | | [2] |
|  |  | NP-2R | agaagacagagttcacagca | 587bp |  |
| TBEV |  | TBEV-R1 | gcgtgytctcckatcactgtca | 1150bp |  |
|  |  | TBEV-F | ggtrytggarctgggdggatg | |  |
|  |  | TBEV-R2 | tcaatrtgmgccacaggaac | 750bp |  |
| SBV |  | SBV-F1 | attgacatgtcaaaatgggggaaagc | 430bp |  |
|  |  | SBV-F2 | aaaaggagtgttggaaccctcttcttcca | 292bp |  |
|  |  | SBV-R | atcaccttgtcagcaccttcttcactgat | |  |
| NSDV |  | NAV-R1 | tagtttgtgctatgctgtgtgttccagc | 380bp |  |
|  |  | NAV-F | cctcaacagggacgagaacaggatcg | | |
|  |  | NAV-R2 | gaagacattgtccttgaagaagtaagac | 336bp |  |
| HTNV |  | HAN-LF1 | atgtaygtbagtgcwgatgc | | [3] |
|  |  | HAN-LR1 | aaccadtcwgtyccrtcatc | |  |
|  |  | HAN-LF2 | gcwgatgchacnaartggtc | |  |
|  |  | HAN-LR2 | gcrtcrtcwgartgrtgdgcaa | 400bp |  |
| WNV |  | WNE-1-445F | accaactactgtggagtc | | [4] |
|  |  | WNE-1-445R | ttccatcttcactctacact | 445bp |  |
|  |  | WEN-2-248F |  |  |  |
|  |  | WEN-2-248R | ccaatgctatcacagacg | 248bp |  |
| JEV |  | JEV-10316-F | graaagaaraytatgtwgaytacatg | | [5] |
|  |  | JEV-10903-R | gatctccyastctattcccaggtgtc | |  |
|  |  | JEV-10483-F-inner | accggatactgrgtagacggtgct | |  |
|  |  | JEV-10770-R-inner | tctcctctaacctctagtccttac | 288bp |  |
| DBSV |  | Da-2-F | atttcacggatgggcttggt | |  |
|  |  | Da-2-R | agcacagccttgacgtagac | |  |
|  |  | Da-5-F | acatccttggtccagatgcg | |  |
|  |  | Da-5-R | atctggtctacccccgagag | 294bp |  |
| ALSV |  | A-M527-F | gacgatcaacaatgctgcgacc | |  |
|  |  | A-M527-R | tagccacttcttcatttgccactcc | 479bp |  |
| JMV |  | 194F1 | tcggcgataaataggagaggtgccat | |  |
|  |  | 194F2 | ggactggagacaagacgtcaacacg | |  |
|  |  | 194R2 | cgccatttcttcatcctccgctag | |  |
|  |  | 194R1 | tctgcgtagagtcggtagaggtggtg | |  |

**Table S2.** Shannon index of virus community.

| **Sample number** | **shannon** |
| --- | --- |
| CMIF | 2.1744066 |
| OSDF1 | 4.10524709 |
| OSDF2 | 4.01715335 |
| CCF | 4.1629304 |
| ISDF | 2.77900372 |
| CMIL | 3.24740441 |
| CCL | 3.9793005 |
| ISDS | 3.9322056 |


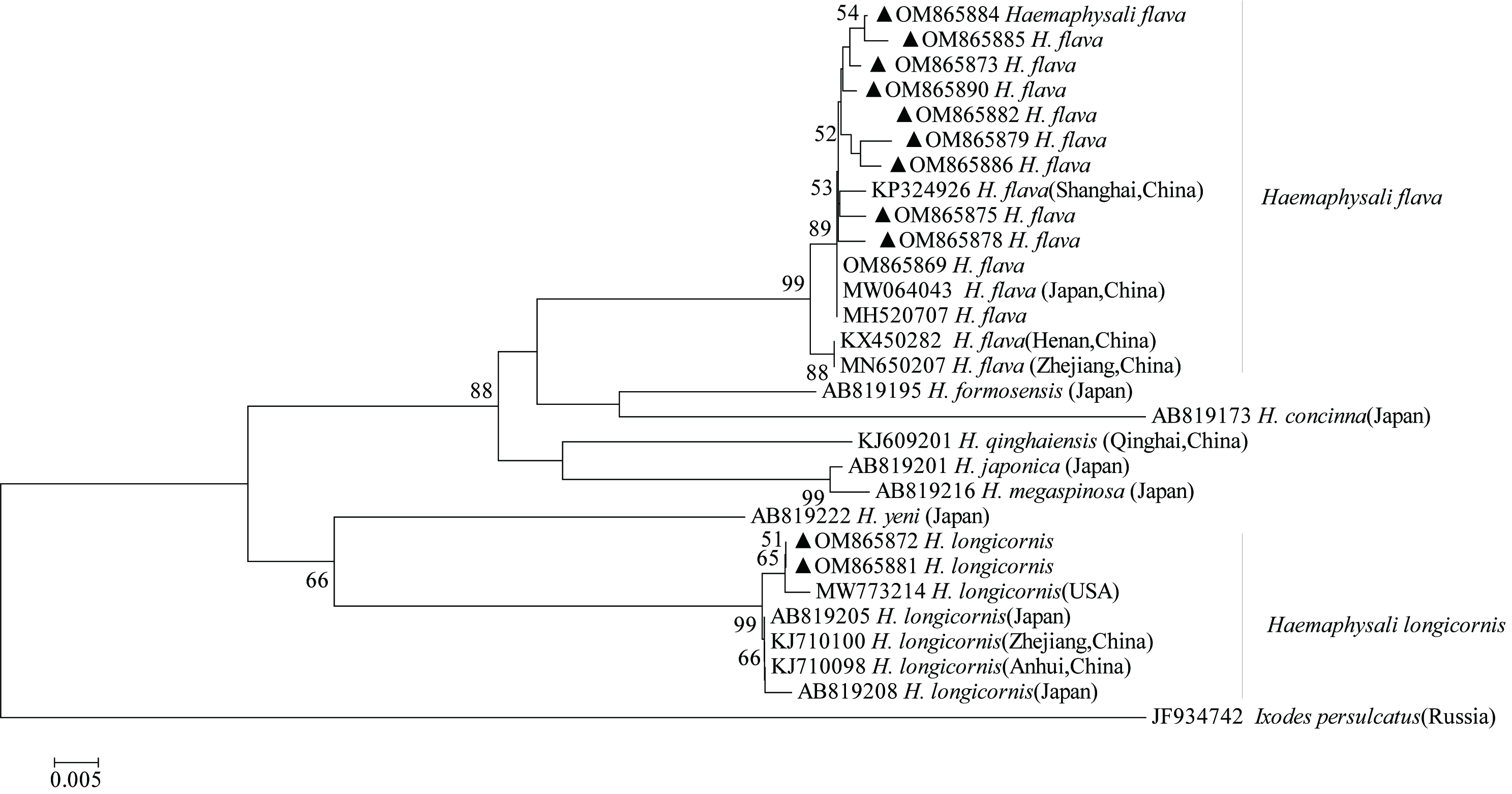


**Figure S1.** Phylogenetic analysis based on *Haemaphysalis* spp. of a fragment (410 bp) of the 16S rDNA gene sequencing and neighbour-joining method (NJ method) (bootstrap replicates 1000 times). Kimura’s two-parameter model was used as a substitution model for the calculation of the phylogenetic trees; ▲ represents the samples from this study.


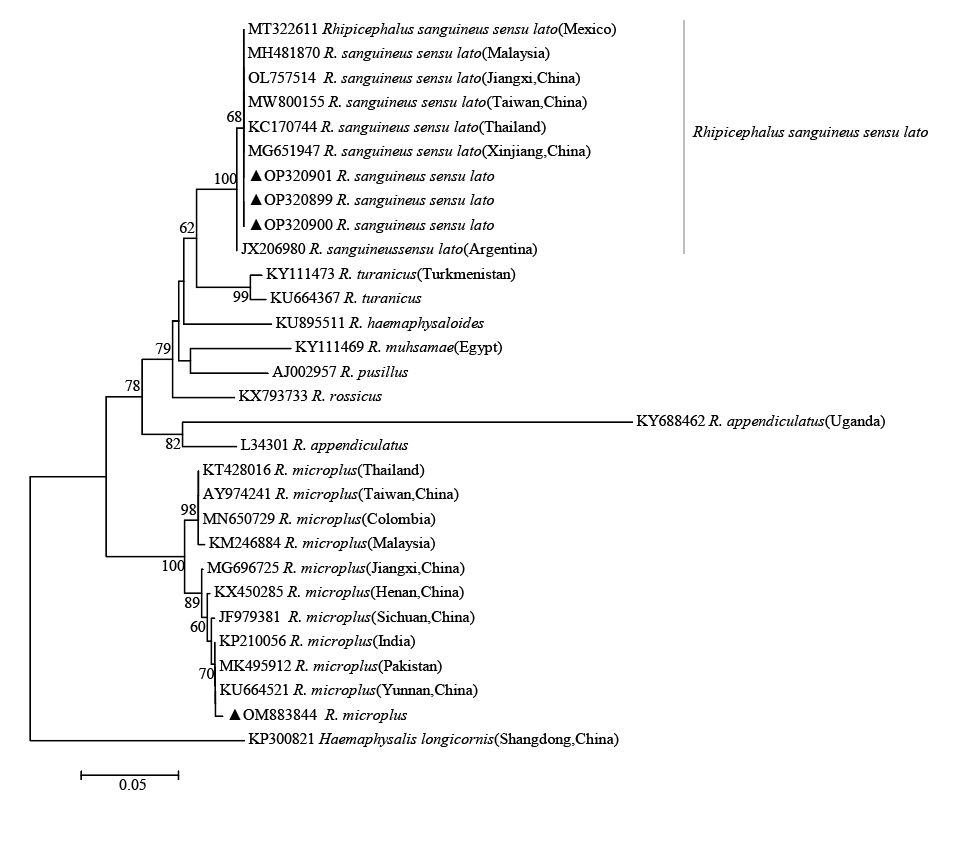


**Figure S2.** Phylogenetic analysis based on *Rhipicephalus* spp. of a fragment (410 bp) of 16S rDNA gene sequencing and neighbour-joining method (NJ method) (bootstrap replicates 1000 times). Kimura’s two-parameter model was used as a substitution model for the calculation of the phylogenetic trees; ▲ represents the samples from this study.


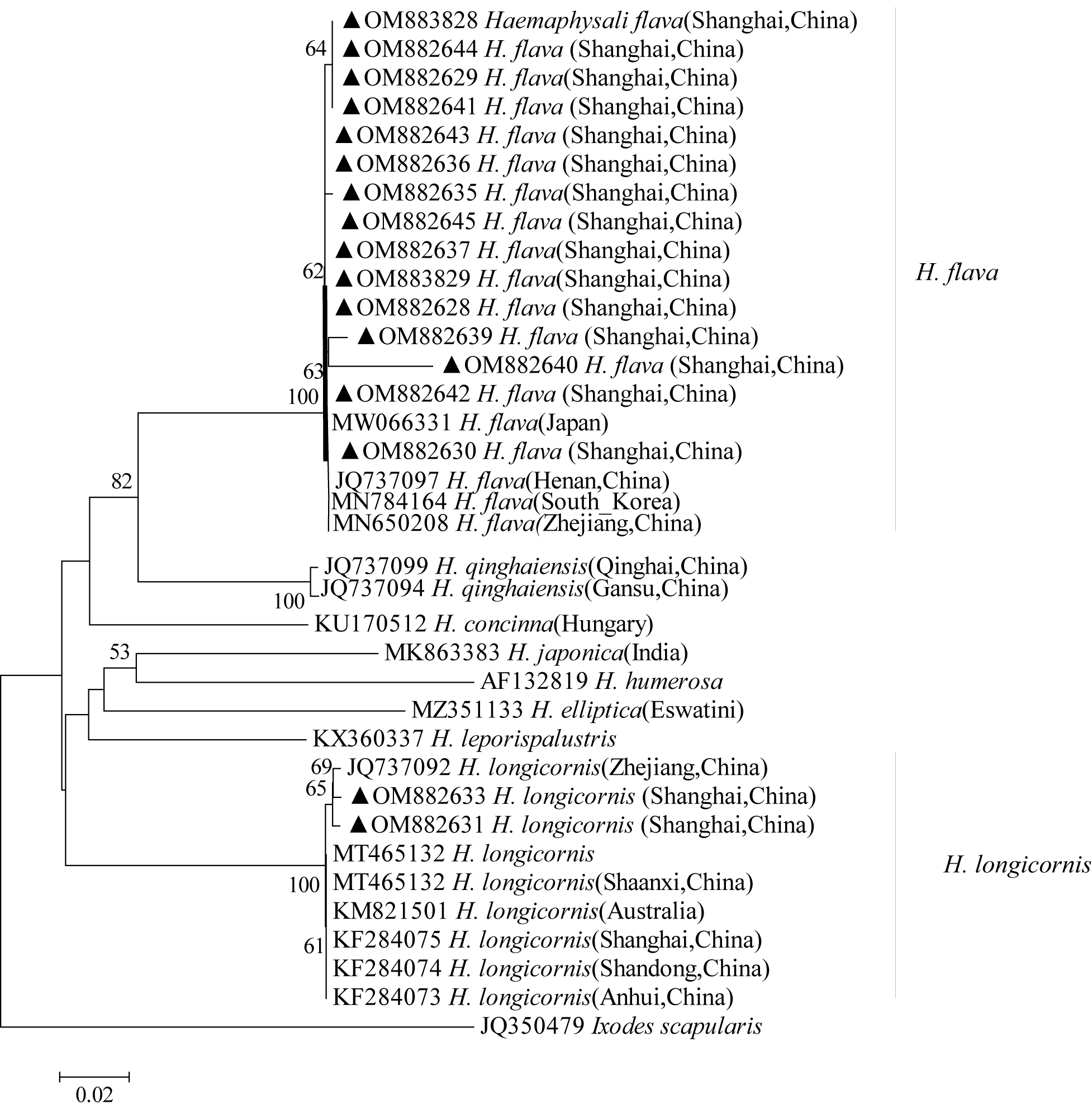


**Figure S3.** Phylogenetic analysis based on *Haemaphysalis* spp. of a fragment (660 bp) of CO I gene sequencing and neighbour-joining method (NJ method) (bootstrap replicates 1000 times). Kimura’s two-parameter model was used as a substitution model for the calculation of the phylogenetic trees; ▲ represents the samples from this study.


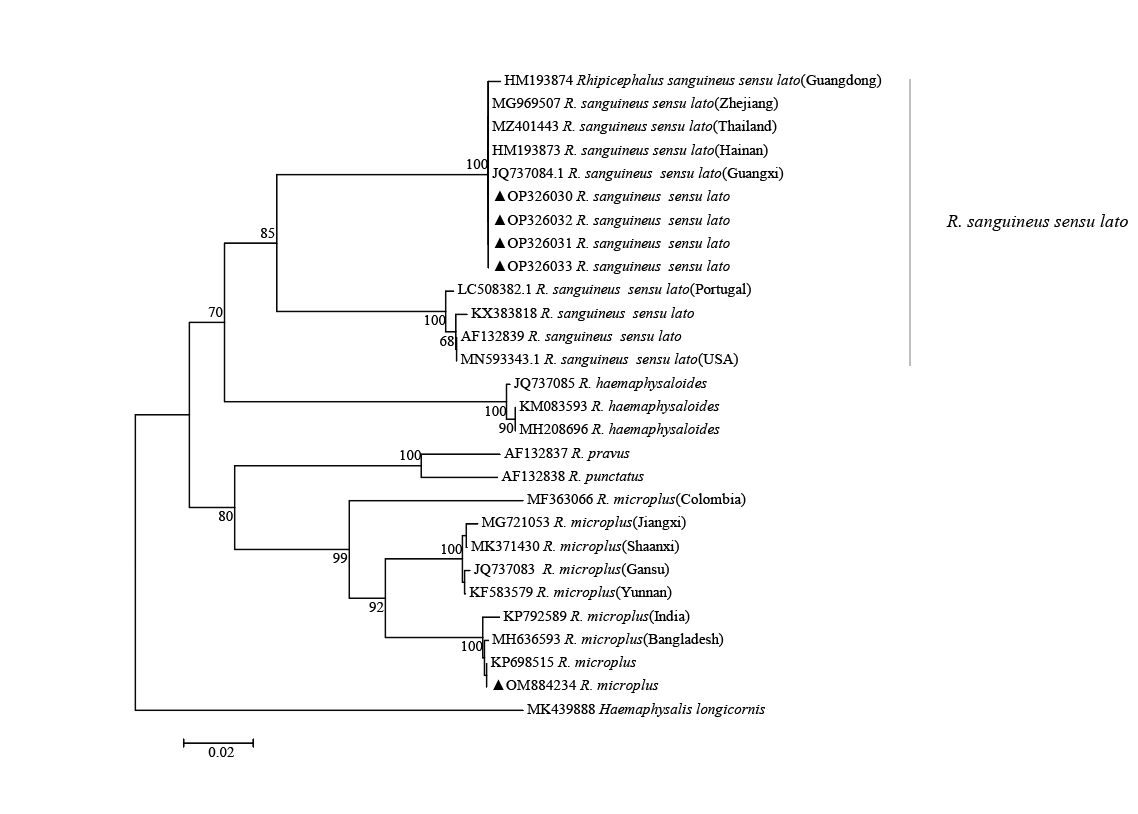


**Figure S4.** Phylogenetic analysis based on *Rhipicephalus* spp. of a fragment (660 bp) of CO I gene sequencing and neighbour-joining method (NJ method) (bootstrap replicates 1000 times). Kimura’s two-parameter model was used as a substitution model for the calculation of the phylogenetic trees; ▲ represents the samples from this study.


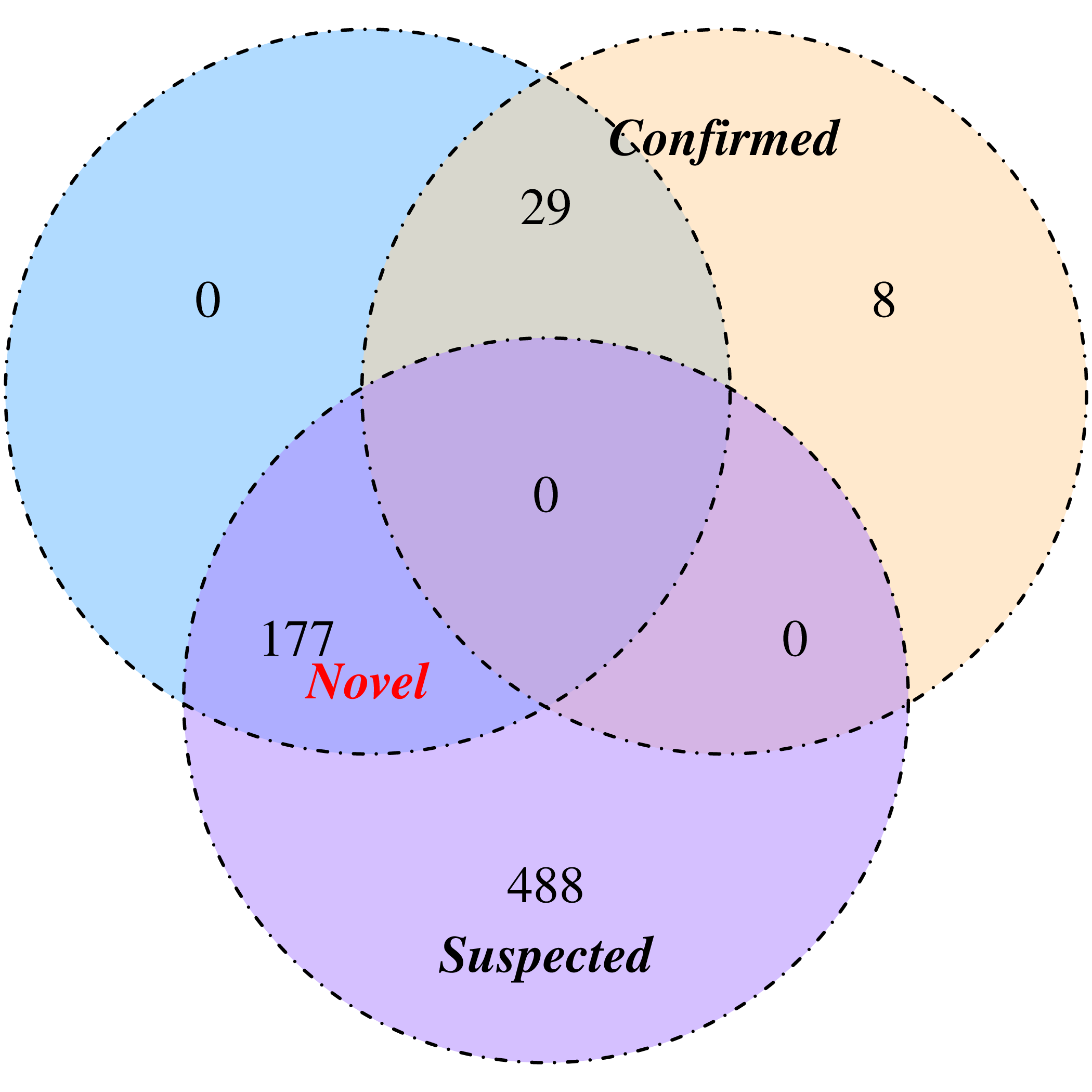


**Figure S5.** Comparison of the number of RNA viruses and unknown virus contigs obtained by different methods.


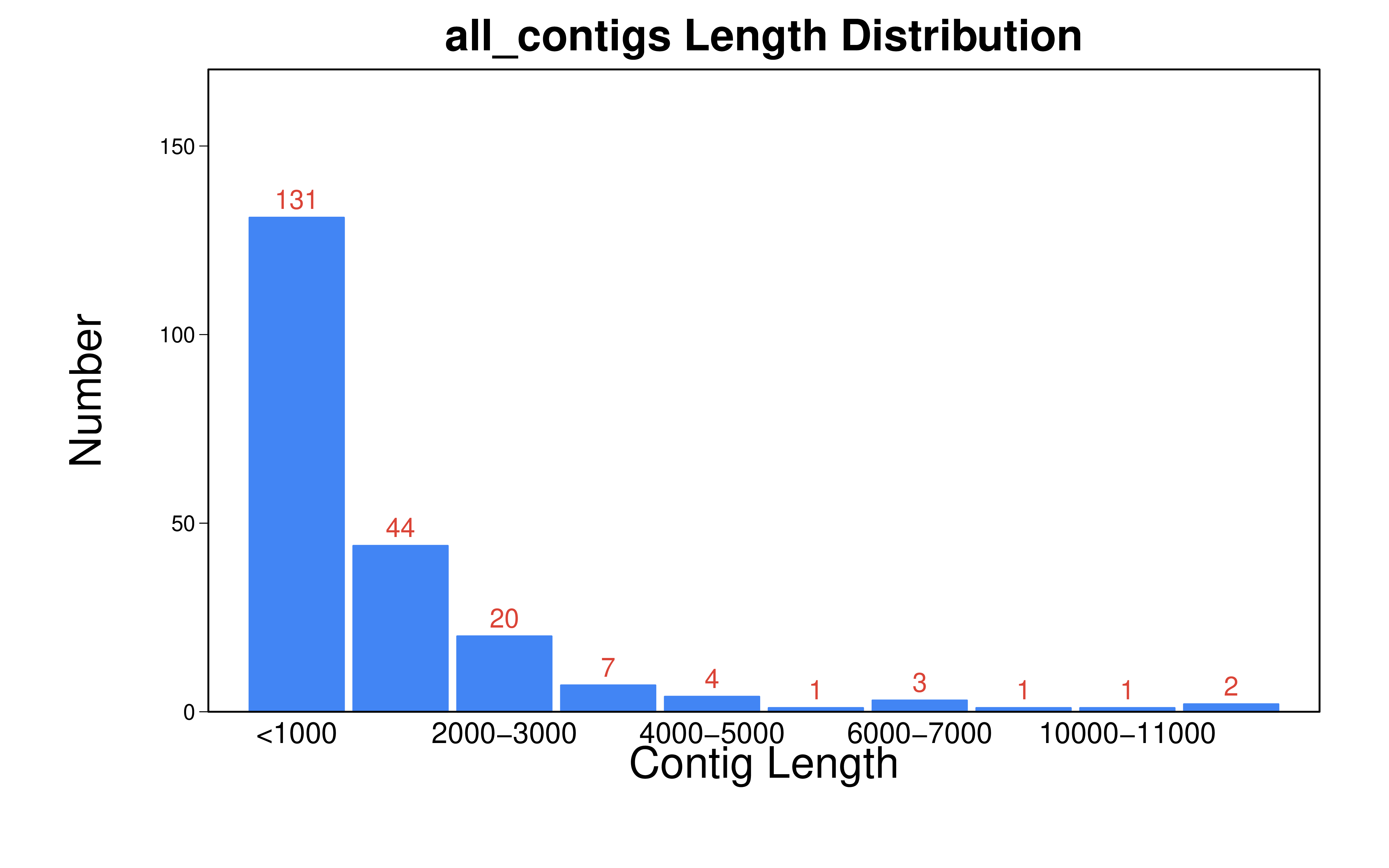


**Figure S6.** contigs length distribution.


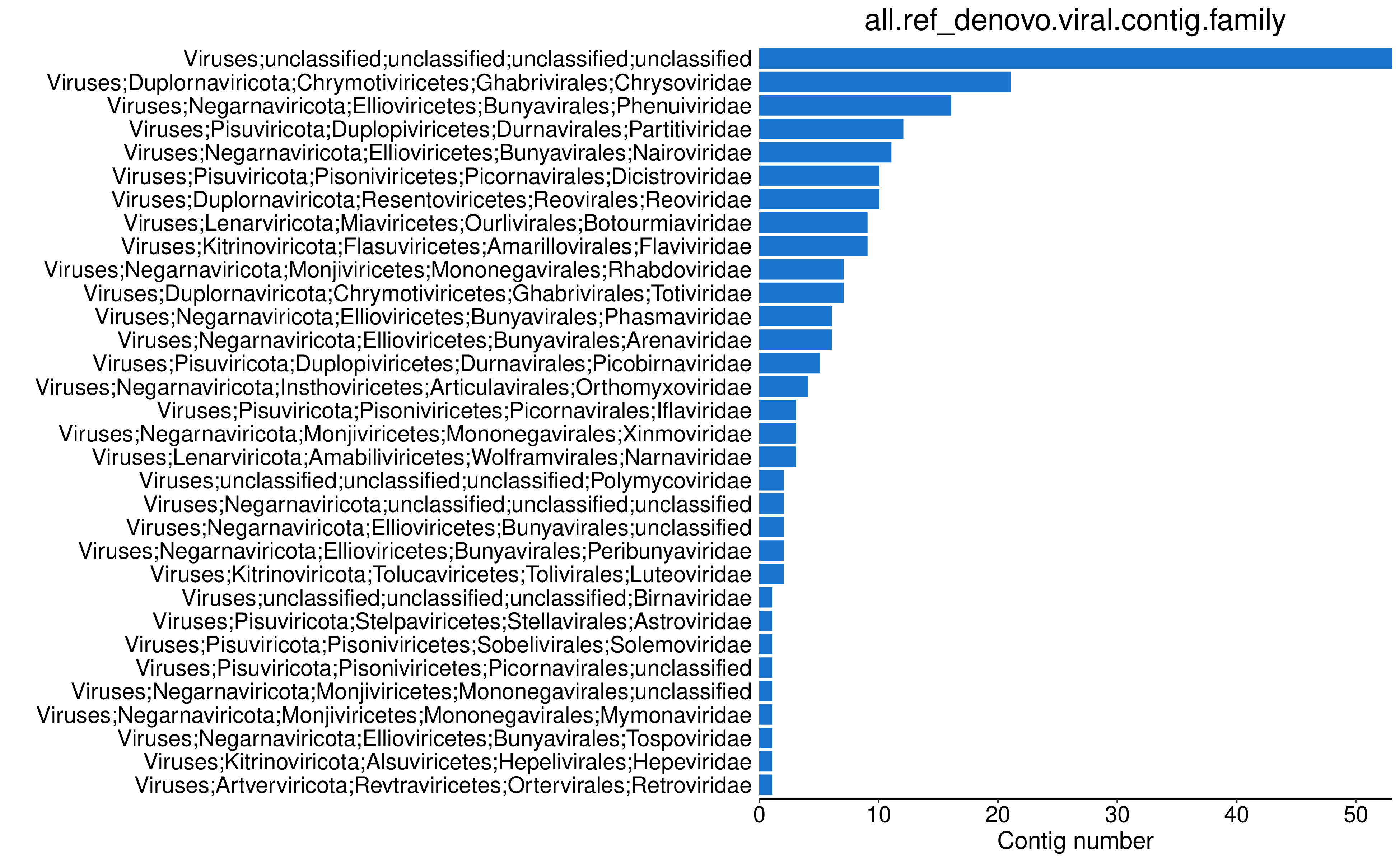


**Figure S7.** RNA virus annotation Statistics based on family level.


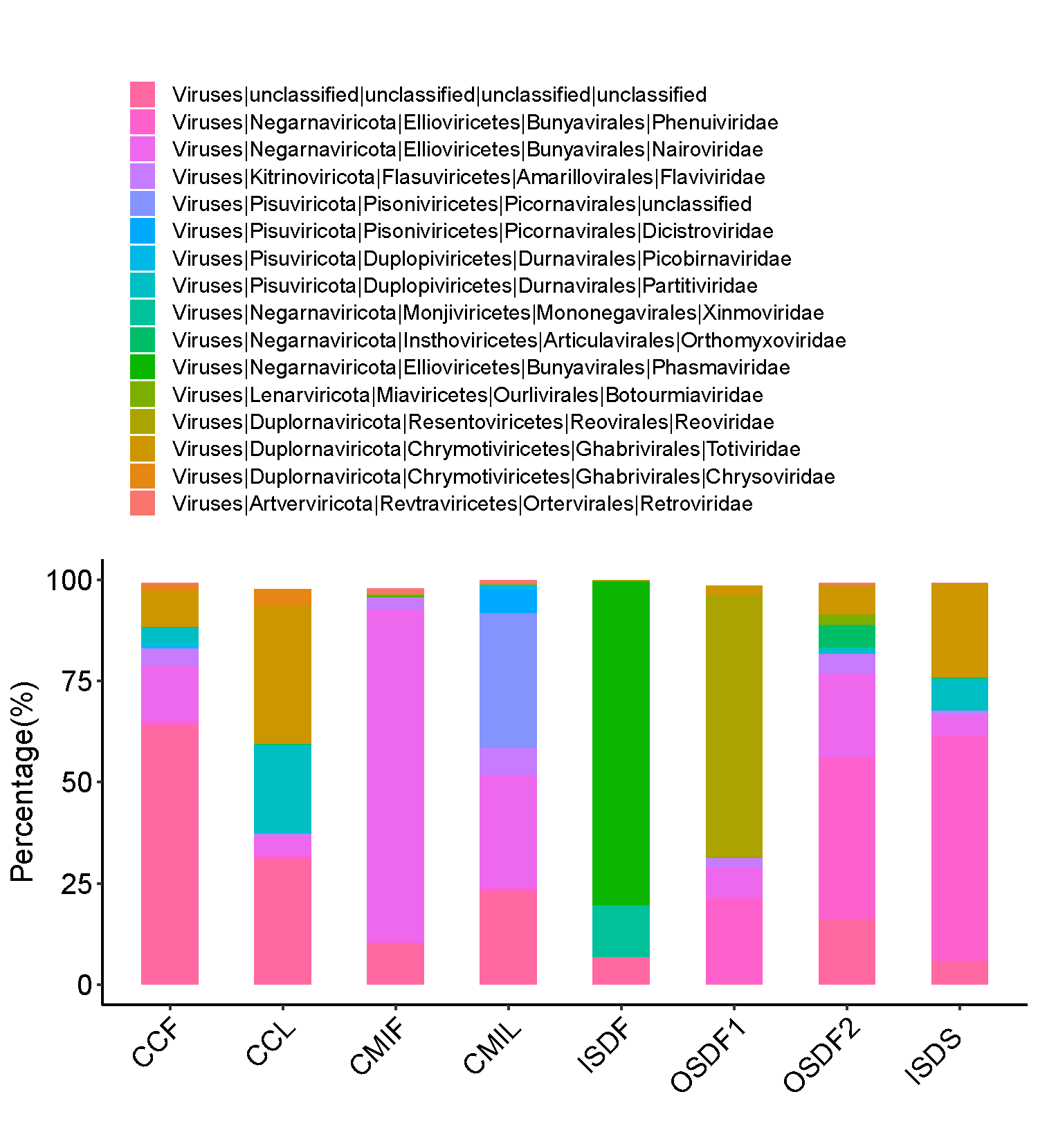


**Figure S8.** RPKM value percentage statistical chart (family level).

*
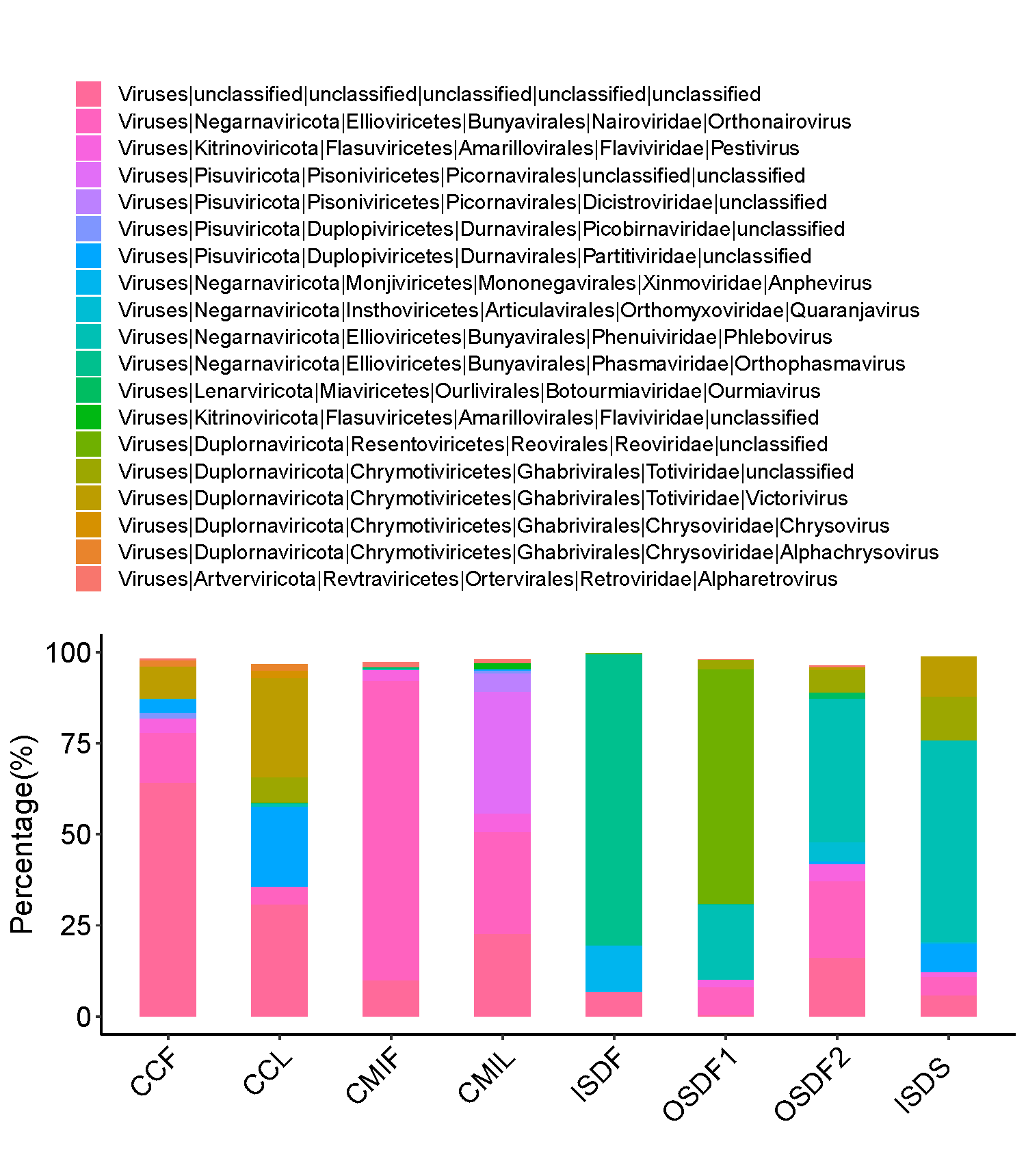
*

**Figure S9.** RPKM value percentage statistical chart (genus level).

*
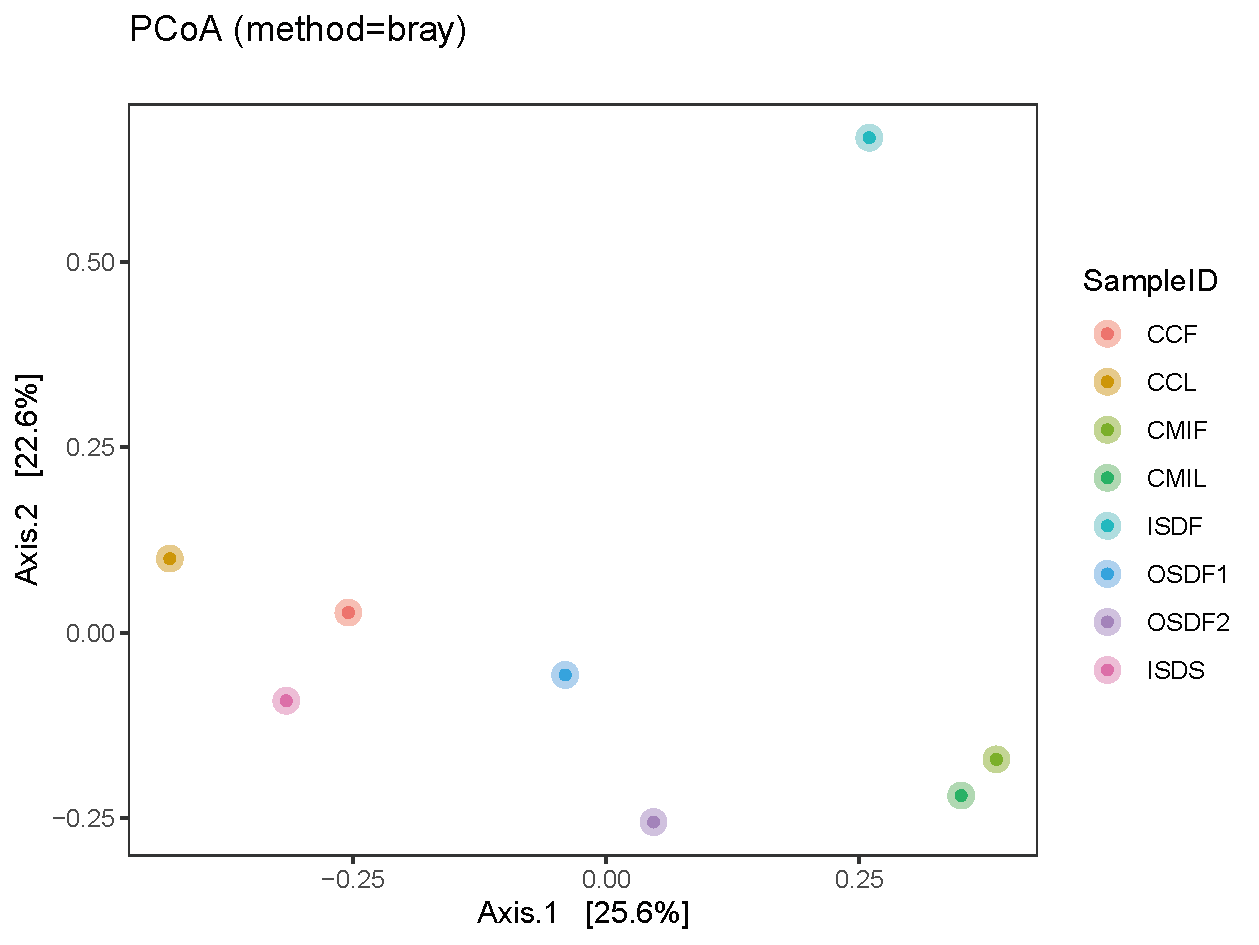
*

**Figure S10.** Principal component analysis (PCA) diagram of ticks captured from different tick species and different locations in Shanghai.

**References**

1. Fakoorziba, M.R.; Naddaf-Sani, A.A.; Moemenbellah-Fard, M.D.; Azizi, K.; Ahmadnia, S.; Chinikar, S. First phylogenetic analysis of a Crimean-Congo hemorrhagic fever virus genome in naturally infected Rhipicephalus appendiculatus ticks (Acari: Ixodidae). *Arch Virol* **2015**, *160*, 1197-1209, doi:10.1007/s00705-015-2379-1.

2. Yoshikawa, T.; Fukushi, S.; Tani, H.; Fukuma, A.; Taniguchi, S.; Toda, S.; Shimazu, Y.; Yano, K.; Morimitsu, T.; Ando, K.; et al. Sensitive and specific PCR systems for detection of both Chinese and Japanese severe fever with thrombocytopenia syndrome virus strains and prediction of patient survival based on viral load. *J Clin Microbiol* **2014**, *52*, 3325-3333, doi:10.1128/jcm.00742-14.

3. Seo, J.W.; Kim, D.Y.; Kim, C.M.; Yun, N.R.; Lee, Y.M.; Lawrence Panchali, M.J.; Kim, D.M. Utility of Nested Reverse-Transcriptase Polymerase Chain Reaction of Clinical Specimens for Early Diagnosis of Hemorrhagic Fever with Renal Syndrome. *Am J Trop Med Hyg* **2021**, *105*, 1285-1289, doi:10.4269/ajtmh.21-0185.

4. Johnson, D.J.; Ostlund, E.N.; Pedersen, D.D.; Schmitt, B.J. Detection of North American West Nile virus in animal tissue by a reverse transcription-nested polymerase chain reaction assay. *Emerg Infect Dis* **2001**, *7*, 739-741, doi:10.3201/eid0704.010425.

5. Swami, R.; Ratho, R.K.; Mishra, B.; Singh, M.P. Usefulness of RT-PCR for the diagnosis of Japanese encephalitis in clinical samples. *Scand J Infect Dis* **2008**, *40*, 815-820, doi:10.1080/00365540802227102.
